# BZD9L1 Differentially Regulates Sirtuins in Liver-Derived Cells by Inducing Reactive Oxygen Species

**DOI:** 10.3390/biomedicines11113059

**Published:** 2023-11-15

**Authors:** Yeuan Ting Lee, Yi Jer Tan, Chern Ein Oon

**Affiliations:** Institute for Research in Molecular Medicine (INFORMM), Universiti Sains Malaysia, Gelugor 11800, Penang, Malaysia; yeuanting93@gmail.com (Y.T.L.); richtan54@gmail.com (Y.J.T.)

**Keywords:** BZD9L1, sirtuin, mitochondrial dysfunction, oxidative phosphorylation, oxidative stress, Agilent Seahorse Cell Mito Stress assay

## Abstract

Growing evidence has highlighted that mitochondrial dysfunction contributes to drug-induced toxicities and leads to drug attrition and post-market withdrawals. The acetylation or deacetylation of mitochondrial proteins can affect mitochondrial functions as the cells adapt to various cellular stresses and other metabolic challenges. SIRTs act as critical deacetylases in modulating mitochondrial function in response to drug toxicity, oxidative stress, reactive oxygen species (ROS), and energy metabolism. We previously showed that a recently characterised SIRT inhibitor (BZD9L1) is non-toxic in rodents in a short-term toxicity evaluation. However, the impact of BZD9L1 on mitochondrial function is unknown. This work aims to determine the effects of BZD9L1 on mitochondrial function in human normal liver and kidney-derived cell lines using the Agilent Seahorse Cell Mito Stress Test to complement our short-term toxicity evaluations in vivo. The Mito Stress assay revealed that BZD9L1 could potentially trigger oxidative stress by inducing ROS, which promotes proton leak and reduces coupling efficiency in liver-derived THLE cells. However, the same was not observed in human kidney-derived HEK293 cells. Interestingly, BZD9L1 had no impact on SIRT3 protein expression in both cell lines but affected SOD2 and its acetylated form at 72 h in THLE cells, indicating that BZD9L1 exerted its effect through SIRT3 activity rather than protein expression. In contrast, BZD9L1 reduced SIRT1 protein expression and impacted the p53 protein differently in both cell lines. Although BZD9L1 did not affect the spare respiratory capacity in vitro, these findings call for further validation of mitochondrial function through assessment of other mitochondrial parameters to evaluate the safety of BZD9L1.

## 1. Introduction

Mitochondria are prominently involved in energy metabolism and cellular processes [1,2,3,4]. Alterations in mitochondrial energy metabolism can result in various pathogenesis and diseases [5,6]. Therefore, it is unsurprising that certain drug treatments and therapeutic interventions may give rise to off-target effects and oxidative stress, thereby impairing mitochondria function and causing significant adverse reactions [5,7]. Drug treatment may impair mitochondrial function at the mitochondrial-regulated biochemical pathways such as the electron transport chain (ETC), ATP synthase, and mitochondrial transporters, as well as at gene transcription and protein translation levels [4]. Mitochondrial bioenergetics plays a significant role in the liver and kidney, which are highly metabolically active organs; thus, understanding these processes is crucial for pharmaceutical interventions. In addition, understanding how drugs impact mitochondrial bioenergetics is essential for predicting drug clearance, potential drug–drug interactions, and drug toxicity. However, the detection of mitochondrial function in rodents remains challenging as most animals used in in vivo toxicity studies are drug naïve, and the effect is primarily idiosyncratic [4,8]. Many efforts have been undertaken to bridge the gap between drug discovery and drug-induced mitochondrial dysfunction. One such approach is by measuring the oxygen consumption rate (OCR) of mitochondria, which is a valuable method for assessing mitochondrial output.

The “Mito Stress Test” kit of the Seahorse Extracellular Flux (XFe) Analyzer (Agilent Technologies) allows for real-time measurement of the OCR and extracellular acidification rate (ECAR). These measurements serve as indicators of mitochondrial function and cellular metabolism [9,10]. The Mito Stress Test entails the sequential injection of three respiration modulators: ATP synthase inhibitor oligomycin A (Oligo), an uncoupler carbonyl cyanide-4-(trifluoromethoxy) phenylhydrazone (FCCP), and ETC complex I and III inhibitors (rotenone/antimycin A) (Rot/AA) [10,11,12]. These modulators collectively enable the evaluation of mitochondrial function. Therefore, the Mito Stress Test could be applied as a preliminary drug screening tool to determine potential drug impact on cellular bioenergetics and mitochondrial function [12].

Sirtuins (SIRTs) are conserved histone deacetylases that rely on NAD^+^ as a cofactor for sensing energy fluctuations associated with metabolic and stress responses [6,13,14]. SIRTs are critical in modulating mitochondrial function in response to oxidative stress, ROS, and energy metabolism [15]. BZD9L1 is a SIRT1 and SIRT2 inhibitor reported to down-regulate SIRT1 and SIRT2 gene and protein expression in healthy and cancer models in vitro and in vivo [16,17,18,19]. In addition, SIRT1 and SIRT3 play a significant role in modulating mitochondrial biogenesis [20,21]. Hence, it is necessary to determine if BZD9L1 affects these SIRT members alongside the assessment of mitochondrial function, as changes in SIRT expression may impact mitochondrial activity and output.

We have previously reported the anti-cancer properties of the BZD9L1 SIRT 1 and 2 inhibitors, which blocked cell proliferation by inducing ROS and apoptosis in colorectal cancer cells [16,17,18]. Moreover, acute and repeated dose toxicity studies also revealed no significant side effects in mice [19]. However, the impact of BZD9L1 on mitochondrial function is unknown and needs to be investigated to complement the animal toxicity study. This work aims to investigate the effects of BZD9L1 on mitochondrial output in the human liver-derived cell line, THLE-2, and kidney-derived cell line, HEK293, using the Agilent Seahorse Cell Mito Stress Test as a preliminary screening tool.

## 2. Materials and Methods

### 2.1. Cell Culture

The human liver-derived cell line THLE-2 and the kidney-derived cell line HEK293 are widely used as in vitro models for pharmaco-toxicological studies [22,23,24]. The THLE-2 cells were purchased from the American Type Culture Collection (ATCC) and cultured in Bronchial Epithelial Cell Growth (BEGM) complete media (Lonza, Morristown, NJ, USA). The BEGM media were supplemented with bovine pituitary extract (BPE), hydrocortisone, human epidermal growth factor (hEGF), insulin, triiodothyronine, transferrin, and retinoic acid from the BEGM Bullet Kit (Lonza) with the addition of 70 ng/mL phosphoethanolamine (Sigma-Aldrich, Burlington, MA, USA) and 10% FBS (Tico Europe, Amstelveen, The Netherlands). HEK293 cells were a kind gift from Prof. Dr. Vikneswaren from Universiti Sains Malaysia, Malaysia. HEK293 cells were cultured in DMEM media (Nacalai Tesque, Kyoto, Japan) supplemented with 10% FBS, 100 units/mL penicillin (Nascalai Tesque) and 100 units/mL streptomycin (Nascalai Tesque). All cell lines were cultured and maintained in 25 or 75 cm^2^ tissue culture-grade flasks. Cells were incubated at 37 °C in a humidified 5% CO_2_ atmosphere.

### 2.2. Cell Treatment

BZD9L1 was dissolved in DMSO (Nacalai Tesque), stored as a 10 mM stock solution aliquot, and kept in the dark at −20 °C. BZD9L1 stock solution was further diluted with cell culture media to prepare a working solution according to the test concentrations.

### 2.3. Cell Viability Assay

The cytotoxicity of BZD9L1 on THLE-2 and HEK293 cell cultures was evaluated with 3-(4,5-dimethylthiazol-2-yl)-2,5-diphenyltetrazolium (MTT) assay (Thermo Fisher Scientific, Waltham, MA, USA). A total of 5.0 × 10^3^ and 2.5 × 10^3^ cells/well of THLE-2 and HEK293 cells were seeded on a 96-well microplate and incubated for 24 h to allow proper cell attachment. On the next day, cells were treated with various concentrations of BZD9L1 with a total volume of 100 µL/well of the treatment media for 24, 48, and 72 h under normal conditions. Each concentration consists of three technical replicates, and the assay was repeated to generate at least three biological replicates. MTT assay was performed immediately at the end of each treatment timepoint. After incubation for 4 h in the dark at 37 °C, the media were removed, and 100 µL DMSO was added to solubilise any formazan crystals formed during the reaction. The quantity of formazan formed was presumably directly proportional to the number of viable cells. It was then measured by recording changes in absorbance at 570 nm using the Tecan Infinite M200 microplate reader (Tecan, Männedorf, Switzerland). Cell viability was determined using the following formula:

Cell viability (%) = (Absorbance of samples)/(Absorbance of vehicle control) × 100%

### 2.4. ROS Assay

The procedure of ROS assay was conducted using the DCFDA/H2DCFDA–Cellular ROS Assay Kit (Abcam, Cambridge, UK) at 6 h as suggested by the manufacturer’s protocol. Briefly, THLE-2 and HEK293 cells were seeded on a clear bottom 96-well microplate with 10,000 cells per well and incubated overnight. The cells were then treated with BZD9L1 for 6 h or *tert*-Butyl-hydroperoxide (tBHP) at 200 µM for 4 h as a positive control for ROS induction. Subsequently, the treatment media was removed and rinsed with 100 µL/well of the 1X Buffer, followed by 100 µL/well of the diluted DCFDA solution. The cells were incubated for 45 min at 37 °C in the dark. The plate was immediately read on a fluorescence plate reader at Ex/Em = 485/535 nm. The relative ROS production was calculated by subtracting the background readings from all measurements and determining fold change with respect to the assay control.

### 2.5. Mito Stress Test Assay

The experiment was conducted according to the Agilent Seahorse XFe Cell Mito Stress Test protocol (Agilent Technologies, Santa Clara, CA, USA). Briefly, a total of 10.0 × 10^3^ cells/well of THLE-2 or HEK293 cells were seeded on the Agilent Seahorse XFe24 Cell Culture Microplates (Agilent Technologies) and incubated overnight to allow proper cell attachment. The cells were then treated with the vehicle control (DMSO) or BZD9L1 at concentrations corresponding to IC_50_ and half IC_50_ for 24 h. Before the assay day, the Agilent Seahorse XFe Analyzer was switched on and left to warm up overnight. The sensor cartridge was hydrated with Seahorse XF Calibrant (Agilent Technologies) and incubated in a non-CO_2_ incubator at 37 °C overnight. On the assay day, the assay medium was prepared by supplementing the Seahorse XF DMEM medium (Agilent Technologies) with 1 mM pyruvate, 2 mM glutamine, and 10 mM glucose (Agilent Technologies). The assay medium was warmed in a water bath at 37 °C before use. Subsequently, the plated cells were rinsed with the warmed assay medium three times to remove the cell growth medium. Each well received a total of 500 µL of the assay media, and the microplate was then incubated at 37 °C in a non-CO_2_ environment for 45 min to 1 h. During the cell incubation time, the Mito Stress Test compounds (1 M oligomycin A, 2 M FCCP, and 0.5 M Rot/AA) (Agilent Technologies) were prepared according to the protocol and were resuspended and diluted with the prepared assay media to reach a final concentration of 100, 100, and 50 µM each. The diluted oligomycin A, FCCP, and Rot/AA were then loaded into the injection ports A, B, and C on the sensor cartridge with volumes of 55, 62, and 69 µL, respectively. After successfully loading the working solutions, the calibration plate and sensor cartridge were loaded into the XFe24 extracellular flux analyser for calibration. When prompted, the calibration plate was replaced with the cell culture microplate, and the assay was initiated. The method and plate setup were preset in the software. The parameters were set as follows: baseline, three cycles; inject port A (Oligo A), three cycles; inject port B (FCCP), three cycles; inject port C (Rot/AA), three cycles. Each cycle comprises periods of mixing (3 min), waiting (0 min), and measuring (3 min).

The cell plate and the sensory cartridges were removed when the run was completed. The cell plate was rinsed with PBS and subjected to the MTT assay. The cell viability data from MTT assay were used to normalise the Mito Stress Test results, as previously described by Little and colleagues [25]. The OCR data were expressed as pmol/min and ECAR as mpH/min. Data for the OCR, ECAR, and mitochondrial respiration parameters such as basal respiration, maximal respiration, proton leak, ATP production, and spare respiratory capacity were generated automatically by the Seahorse XF Mito Stress Test Report Generator.

### 2.6. Western Blot

The whole-cell extract after treatment was collected after resuspending in ice-cold 8 M urea lysis buffer and stored at −20 °C. The protein concentration was quantified using the bicinchoninic acid protein assay (Nacalai Tesque). Approximately 30 μg of protein was loaded and run on 10% sodium dodecyl sulfate (SDS) polyacrylamide gel and then transferred to a polyvinylidene fluoride membrane. Following a step of blocking in 5% milk in 1% Tween 20-buffered Tris-saline, the primary antibodies specific to the desired proteins were incubated with the membrane. The primary antibodies were SIRT1 (Cell Signaling Technology, Danvers, MA, USA, 2496S, rabbit polyclonal), SIRT3 (Cell Signaling Technology, 5490, rabbit polyclonal), SIRT4 (Santa Cruz, Santa Cruz, CA, USA, sc-135797, mouse monoclonal), SIRT5 (Santa Cruz, sc-271635, mouse monoclonal), SOD2 (acetyl K68) (Abcam, ab137037, rabbit polyclonal), SOD2 (GeneTex, Irvine, CA, USA, GTX116093, rabbit polyclonal), p53 (Cell Signaling Technology, 9282S, rabbit polyclonal), acetylated p53 (acetyl K382) (Abcam, ab75754, rabbit polyclonal), PARP (Cell Signaling Technology, 9542, rabbit polyclonal), β-actin (GeneTex, GTX109639, rabbit polyclonal), at 4 °C overnight. After washing the membrane with TBST washing solution three times, 10 min each, the membrane was incubated with diluted HRP-conjugated secondary antibodies (anti-mouse IgG, HRP-linked antibody, Cell Signaling Technology, 7076; anti-rabbit IgG antibody, Cell Signaling Technology, 7074) in blocking solution at room temperature for 1 h. Protein bands were visualised by incubating the membrane with Chemi-Lumi One Super (ECL) solution (Nacalai Tesque) in the dark for 1 min and scanned using the C-DiGit^®^ Blot Scanner (LI-COR Bioscience, Lincoln, NE, USA). Densitometric analysis of band intensity for each blot was performed using the Image Studio™ Lite software version 5.2.5 (LI-COR Bioscience) and normalised to β-actin loading control.

### 2.7. Statistical Analyses

GraphPad Prism 8.0 (GraphPad, San Diego, CA, USA) was used to analyse all data. The analysis of variance (ANOVA) test was employed to compare mean values among three or more data sets, and Bonferroni’s post-test was used to compare any two data sets among the three or more sets. Statistical significance was indicated in the figures by *, *p* < 0.01, ***, *p* < 0.001, and **** *p* < 0.0001, compared to vehicle control. All error bars depict the standard error of the mean (SEM).

## 3. Results

### 3.1. BZD9L1 Reduced THLE-2 and HEK293 Cell Viability but Induced ROS Only in THLE-2 Cells

The effect of BZD9L1 (0–200 µM) on human liver-derived THLE-2 cells and kidney-derived HEK293 cells was assessed using the MTT assay at 24, 48, and 72 h. The IC_50_ for each cell line is listed in Table 1.

**Figure 1:**
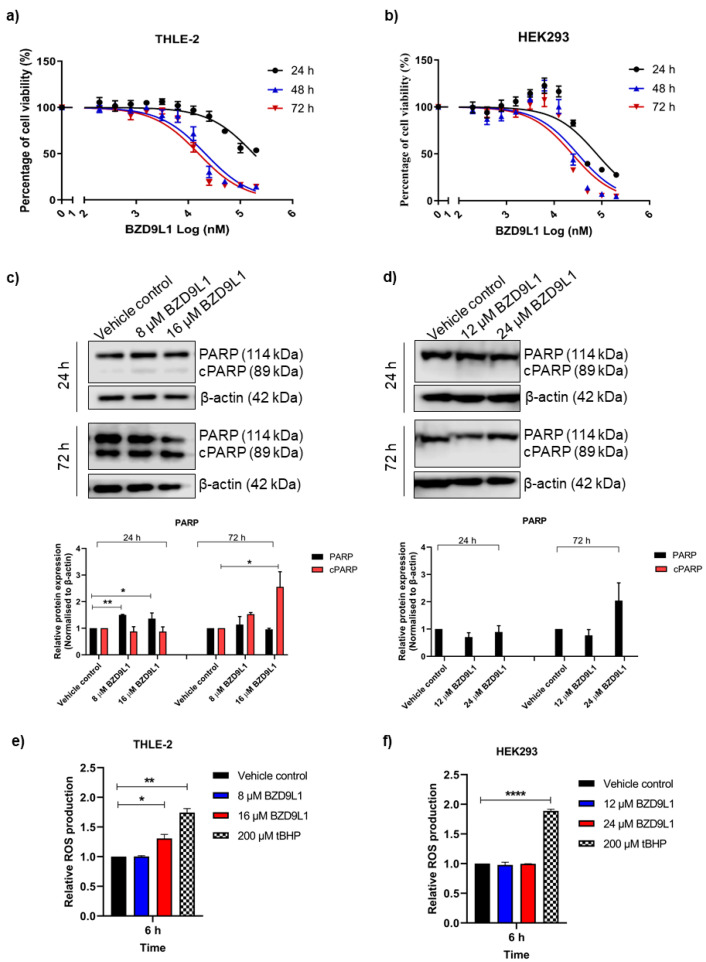
BZD9L1 reduced the metabolic viability of THLE-2 and HEK293 cells but induced ROS levels only in THLE-2 cells. Representative graphs demonstrating the cell viability of (**a**) THLE-2 and (**b**) HEK293 cells post-BZD9L1 treatment at 24, 48, and 72 h. Representative blots showing the protein levels of PARP and cPARP in (**c**) THLE-2 and (**d**) HEK293 whole-cell lysates post BZD9L1 treatment for 24 and 72 h, *n* = 3 independent experiments. cPARP was not detected across all treatments in HEK293 cells. β-actin was used as a loading control. Densitometry analysis of band intensity for each blot was performed using Image Studio^TM^ Lite software, and the resulting values were normalised to the corresponding level of β-actin. ROS levels were measured using the DCFDA assay in human (**e**) THLE-2 and (**f**) HEK293 cells. Cells were pretreated with BZD9L1 at the indicated concentrations for 6 h. *tert*-Butyl-hydroperoxide (tBHP) at 200 µM was used as a positive control. All graphs report means ± SEM (error bars) of triplicates. Statistical analysis (* *p* < 0.05, ** *p* < 0.01, **** *p* < 0.0001, one-way ANOVA, Bonferroni’s post-test) was determined using GraphPad Prism 8.0. Statistical significance (*) depicts a comparison between the treatment group and the vehicle control.

BZD9L1 reduced THLE-2 and HEK293 cell viability in a time- and dose-dependent manner (Figure 1a,b). The IC_50_ and half-IC_50_ concentration of BZD9L1 in both cell lines at the 72 h time point (i.e., 16 and 8 µM for THLE-2; 24 and 12 µM for HEK293 cells, respectively) were employed in downstream Mito Stress assay and Western blot analysis to determine the effect of BZD9L1 at non-cytotoxic doses on mitochondrial functions and early expression of SIRT proteins at 24 h. At both concentrations, BZD9L1 demonstrated a marginal increase in PARP protein expression but had no effect on cleaved PARP (cPARP) compared to vehicle control in THLE-2 cells 24 h post-treatment (Figure 1c). However, BZD9L1 at 16 µM increased cPARP expression marginally compared to vehicle control at 72 h (Figure 1c). Interestingly, HEK293 cells demonstrated no significant change in PARP protein expression and no signs of apoptotic cell death at both doses and treatment periods with undetectable cPARP protein expression (Figure 1d). A marginal but significant induction of ROS was observed in THLE-2 cells treated with 16 µM BZD9L1 at 6 h (Figure 1e). On the other hand, no ROS induction was detected in HEK293 cells (Figure 1f).

### 3.2. Effects of BZD9L1 on the Bioenergetics Profile of THLE-2 and HEK293 Cells

**Figure 2:**
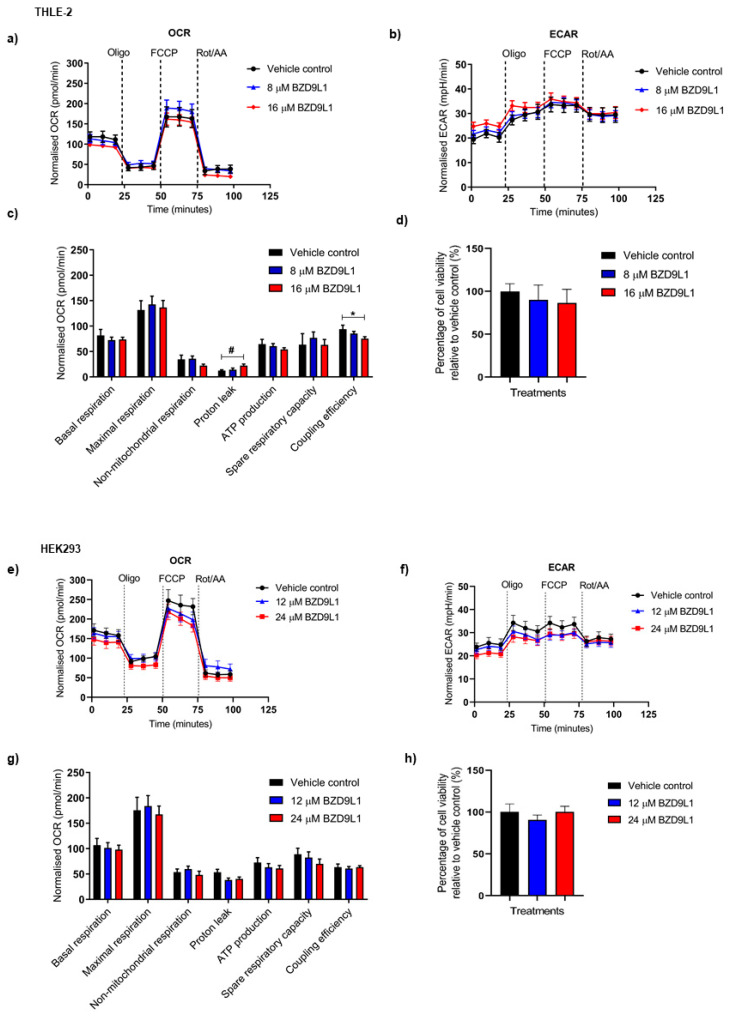
The effect of BZD9L1 on oxygen consumption rate (OCR) and extracellular acidification rate (ECAR) in (**a**–**d**) THLE-2 and (**e**–**h**) HEK293 cells 24 h post-treatment with BZD9L1. The (**a**,**e**) OCR and (**b**,**f**) ECAR rates in THLE-2 and HEK293 cells were measured using the Seahorse XF analyser and were normalised to MTT optical density at the endpoint of the assay. (**c**,**g**) Graphs show the mitochondrial respiration parameters in THLE-2 and HEK293 cells, including basal respiration, maximal respiration, non-mitochondrial respiration, proton leak, ATP production, spare respiratory capacity, and coupling efficiency. (**d**,**h**) MTT assay of THLE-2 and HEK293 cells following the Mito Stress Test showed no significant difference in cell viability. The graphs report means ± SEM (error bars, *n* = 4 independent experiments). Statistical analysis (* *p* < 0.05, one-way ANOVA, Bonferroni’s post-test; # *p* < 0.05, Student’s *t*-test) was determined using GraphPad Prism 8.0. Statistical significance (*) depicts the comparison between the treatment group and vehicle control. Oligo, oligomycin A; FCCP, carbonyl cyanide-p-trifluoromethoxyphenylhydrazone, Rot/AA, rotenone, and antimycin A.

Given the crucial role of mitochondrial respiratory electron transport in energy metabolism and ROS production, the effects of BZD9L1 treatment on mitochondrial function were evaluated. The bioenergetic profiles of THLE-2 and HEK293 cell lines treated with BZD9L1 at their respective doses for 24 h are presented in Figure 2. BZD9L1 did not affect the basal OCR and ECAR at both concentrations in THLE-2 cells (Figure 2a,b). However, BZD9L1 at 16 µM significantly increased proton leak and reduced coupling efficiency in THLE cells (Figure 2c). No additional notable alterations were detected in other mitochondrial parameters. In HEK293 cells, BZD9L1 exhibited no significant changes in OCR, ECAR, and individual mitochondrial parameters (Figure 2e–g). The viability of THLE-2 and HEK293 cells remained unaffected by the drug treatments after the Mito Stress Test assay at 24 h, as validated by the MTT assay (Figure 2d,h).

### 3.3. Effects of BZD9L1 on the Regulation of Sirtuins in THLE-2 and HEK293 Cells

**Figure 3:**
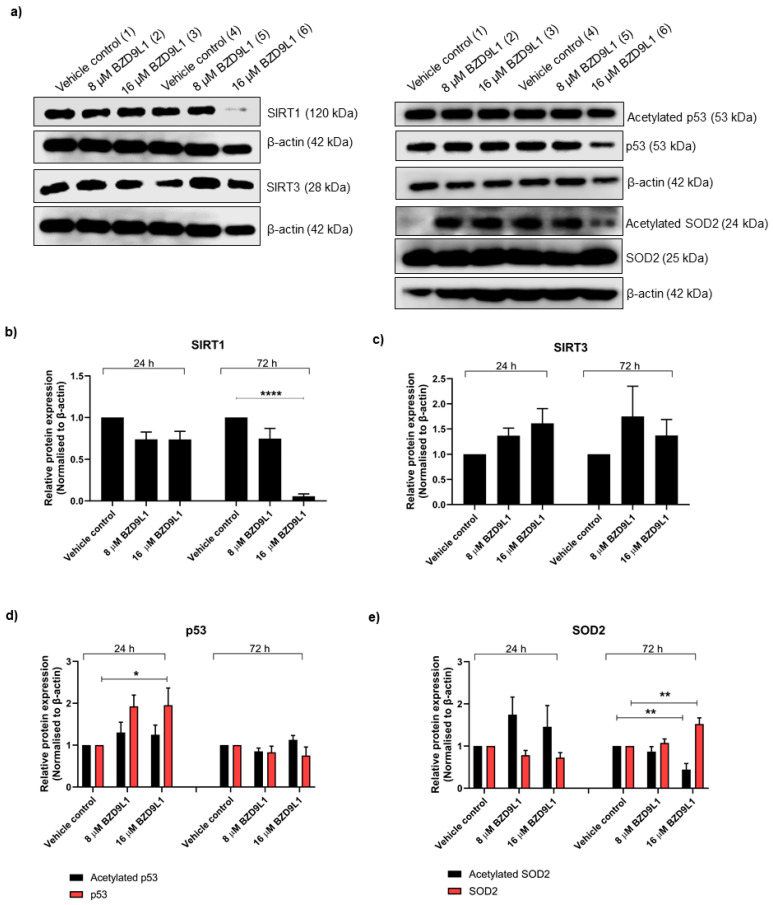
Sirtuin and SOD2 protein expression profile in THLE-2 cells treated with BZD9L1 (**a**–**e**). Representative blots showing protein levels of mitochondrial SIRT1, SIRT3, p53, and SOD2 in THLE-2 whole-cell lysates post BZD9L1 treatment for 24 (lane 1–3) and 72 h (lane 4–6), *n* = at least 3 independent experiments. β-actin was used as a loading control. Densitometry analysis of band intensity for each blot was performed using Image Studio^TM^ Lite software, and the resulting values were normalised to the corresponding level of β-actin. All graphs report means ± SEM (error bars) of triplicates. Statistical analysis (* *p* < 0.05, ** *p* < 0.01, **** *p* < 0.0001, one-way ANOVA, Bonferroni’s post-test) was determined using GraphPad Prism 8.0. Statistical significance (*) depicts a comparison between the treatment group and the vehicle control.

The effect of BZD9L1 on SIRT protein expression was determined at 24 and 72 h to ascertain the potential differential regulation of these proteins at early and late time points. At 16 μM for 72 h, BZD9L1 significantly reduced SIRT1 protein level in THLE-2 cells (Figure 3a,b). The p53 protein is one of the significant deacetylated targets of SIRT1 [26]. Interestingly, down-regulation of SIRT1 at 72 h did not increase the acetylated p53/beta-actin protein ratio compared to vehicle control (Figure 3a,d). A slight increase in the p53/beta-actin ratio was noted in cells treated with 16 μM BZD9L1 at 24 h. However, no alteration in the p53/beta-actin ratio was detected post treatment with BZD9L1 compared to vehicle control at 72 h (Figure 3a,d). The expression level of acetylated superoxide dismutase 2 (SOD2) and SIRT3 proteins remained unchanged compared to the control group at 24 h (Figure 3a,c,e). At 16 µM for 72 h, BZD9L1 diminished the level of acetylated SOD2 but significantly increased the protein expression of SOD2 (Figure 3a,e). Interestingly, SIRT4 and SIRT5 proteins were not detected in THLE-2 cells (Supplementary Figure S1a).

**Figure 4:**
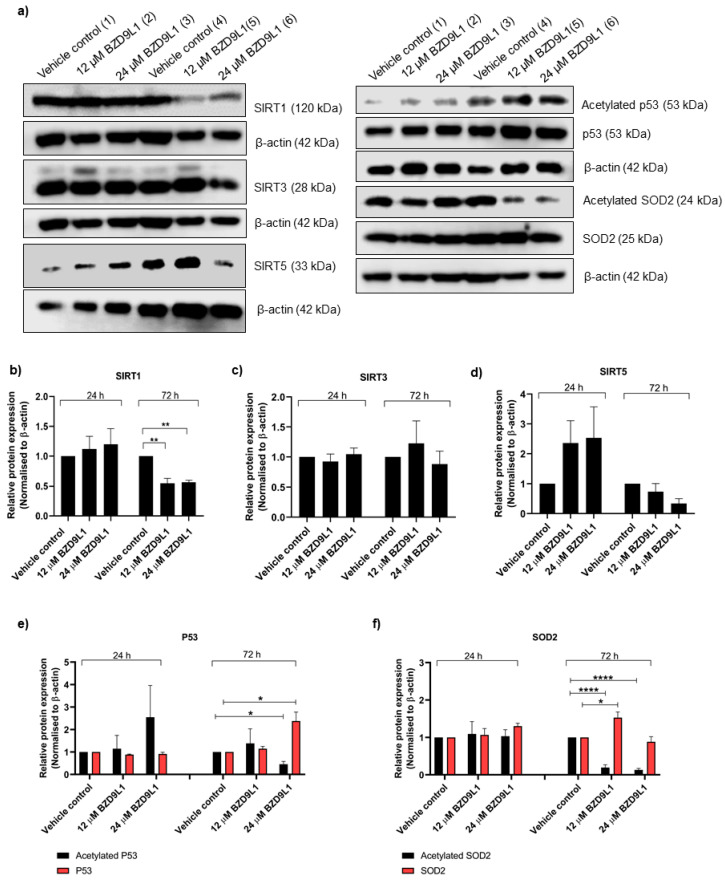
Mitochondrial sirtuin and SOD2 protein expression profile in response to BZD9L1 treatment in HEK293 cells (**a**–**f**). Representative Western blots showing protein levels of SIRT1, SIRT3, SIRT5, p53, and SOD2 in HEK293 cells post BZD9L1 treatment for 24 (lane 1–3) and 72 h (lane 4–6), *n* = at least 3 independent experiments. β-actin was used as a loading control. Densitometry analysis of band intensity for each blot was performed using Image Studio^TM^ Lite software, and the resulting values were normalised to the corresponding level of β-actin. All graphs report means ± SEM (error bars) of triplicates. Statistical analysis (* *p* < 0.05, ** *p* < 0.01, **** *p* < 0.0001, one-way ANOVA, Bonferroni’s post-test) was determined using GraphPad Prism 8.0. Statistical significance (*) depicts a comparison between the treatment group and the vehicle control.

In HEK293 cells, only SIRT3 and SIRT5 proteins, but not SIRT4, were detected at 24 and 72 h post BZD9L1 treatment (Figure 4 and Supplementary Figure S1b). At 12 and 24 μM, BZD9L1 did not affect SIRT3 and SIRT5 protein expression (Figure 4a,c,d). Both 12 and 24 μM BZD9L1 notably reduced the SIRT1/beta-actin ratio at 72 h compared to vehicle control (Figure 4a,b). An increase in the p53/beta-actin ratio and a decrease in acetylated p53/beta-actin ratio were observed in cells treated with 24 μM BZD9L1 at 72 h (Figure 4a,e). Surprisingly, the protein level of acetylated SOD2 was significantly reduced 72 h post-treatment (Figure 4a,f). However, 12 μM BZD9L1 at 72 h increased the expression of the endogenous SOD2 protein compared to vehicle control (Figure 4a,f).

## 4. Discussion

Mitochondrial function has lately garnered increasing attention as one of the critical parameters in drug safety assessment. This is due to its potential correlation with the aetiology of drug-induced organ toxicity [27,28]. In the present study, an increase in proton leak and reduced coupling efficiency without a significantly reduced ATP production may implicate drug-induced oxidative stress [29,30]. The proton leak is a process where H^+^ ions are returned from mitochondrial intermembrane space to the mitochondrial matrix without passing through ATP synthase, resulting in incomplete ATP synthesis. The use of oligomycin in the assay could potentially increase mitochondrial membrane potential, leading to an underestimation of ATP-linked OCR and an overestimation of proton leak [31]. Independent measurement of mitochondrial membrane potential is, therefore, necessary to delineate the mode of action of BZD9L1 on mitochondrial function. Coupling efficiency describes the amount of O_2_ required to fully drive ATP synthesis compared with O_2_ driving proton leak, where reduced coupling efficiency may indicate reduced mitochondrial respiration efficiency and lower ATP production [32]. However, other studies also reported that proton leak induction may not affect the overall energy efficiency in cells [33,34]. BZD9L1 induced oxidative stress through ROS production (measured at 6 h), which enhanced proton leak and reduced coupling efficiency. An increase in proton leak may indicate either an increase in uncoupling protein activity, damage to the inner mitochondrial membrane and/or ETC complexes, or electron slip resulting in increased oxygen consumption without proton translocation [33,35,36]. Thus, the association of BZD9L1-induced proton leak with mitochondria damage warrants further investigation. Furthermore, the spare respiratory capacity determines mitochondrial fitness and reflects the “health” of the mitochondrion [37,38]. The spare respiratory capacity may be deployed to offset the proton leak triggered by the FCCP [37]. No statistical significance was achieved between the BZD9L1 and vehicle control-treated cells, suggesting healthy mitochondrial function [39].

ECAR can be used to study how cells respond to drugs that affect their glycolytic capacity. Drugs that impair mitochondrial function usually accelerate glycolysis in cells to minimise oxidative damage and suppress apoptosis [40,41]. In turn, this may reduce the pH of the culture medium through elevated lactate production, which can be detected as an increase in the ECAR [40,41]. The status of ECAR generation (via glycolysis or CO_2_ production from the tricarboxylic acid cycle (TCA) cycle) was determined through Rot/AA injection [39,42]. A reduction of ECAR upon Rot/AA injection indicated that ECAR is generated from the TCA cycle, while a constant or increased ECAR in response to Rot/AA treatment may indicate ECAR generation via glycolysis [39,42]. Given that both THLE-2 and HEK293 cell lines demonstrated a decrease of ECAR, which remained higher than the basal ECAR in response to Rot/AA injection, the ECAR kinetic may be modestly attributable to CO_2_ production from the TCA cycle as described by Divakaruni and colleagues [42]. Nevertheless, a rigid conclusion on ECAR cannot be made as the Mito Stress Test assay only allows for qualitative interpretation of cellular ECAR. Although the MTT assay has previously been reported as a normalizing tool for the Mito Stress Test assay [25], it is noteworthy that normalising the OCR and ECAR data to MTT readout may result in discrepancies when correlating cellular ATP to the absolute cell numbers [43,44], hence the limitation of this study. Therefore, cell viability assays that do not interfere with cell metabolism may be used as an alternative approach [44].

In general, SIRT3 activates while SIRT4 represses mitochondrial enzymes. Remarkably, SIRT5 activates and represses the mitochondrial enzymes [45]. In the present study, only SIRT3 protein was detected in THLE-2 cells. Furthermore, SIRT3 and SIRT5 but not SIRT4 proteins were detected in HEK293 cells, which is consistent with the findings by others [45,46]. However, the lack of SIRT4 and SIRT5 protein expression in THLE-2 cells or SIRT4 in HEK293 cells contrasted our previous in vivo findings where all mitochondrial SIRTs were found to be expressed in both mouse liver and kidney tissues [19], suggesting the limitation of using cell lines as the microenvironment may be crucial in impacting treatment outcomes. THLE-2 cells express the phenotypic characteristic of normal adult liver epithelial cells, closely related to human hepatocytes [22,47]. However, THLE-2 cells expressed a relatively low level of drug transporters compared to other liver carcinoma cell lines (e.g., HepG2, Hep3B, SK-Hep-1, or Huh7) and human hepatocytes; they also have a lower mRNA expression of NADPH cytochrome P450 (CYP) reductase [22,24,47]. These properties could retain drugs in the cells and increase cellular susceptibility to the cytotoxic effect of BZD9L1. Consequently, this may explain the low IC_50_ value of BZD9L1 in THLE-2 cells.

Alteration of SIRT3 activity in response to cellular environment changes or interference by external factors (e.g., treatment or genetic modification) can affect ATP production and mitochondrial quality [20,48,49,50]. However, no significant changes in SIRT3 protein expression were observed in both cell lines at 24 h post treatment, indicating that BZD9L1 might not affect the SIRT3-ETC complex activities during OXPHOS, which is consistent with the normal ATP production observed in the Mito Stress Test assay. Moreover, ETC is a major site for producing ROS. ROS is the by-product of mitochondrial respiration that contributes to oxidative stress, proton leak, and mitochondrial dysfunction [51]. Excess ROS generated during OXPHOS may lead to DNA damage and impair cell viability [20]. We previously demonstrated the potential of BZD9L1 to induce ROS and activate the mitochondrial apoptotic pathway in colorectal cancer cells [17,18]. BZD9L1 induced ROS and SOD2 protein expression but reduced acetylated SOD2 protein in both THLE-2 and HEK293 cells. SIRT3 is an important regulator in the mitochondrial ROS scavenging pathway via direct deacetylation of mitochondrial SOD2, which is also known as manganese superoxide dismutase (MnSOD) [52,53,54]. Although increased SIRT3 expression was shown to enhance SOD2-mediated ROS reduction, this effect may be reversed upon SIRT3 depletion [52]. Hence, increased ROS production may be essential to trigger the deacetylate activity of SIRT3 to activate the SOD2 protein, resulting in the conversion of superoxide into hydrogen peroxide and water [33,52,55]. On the other hand, acetylated SOD2 may lose its dismutase activity and be further converted to pro-oxidant (peroxidase) macromolecules that can cause unspecific substrate oxidation and oxidative damage [56]. Reduction of acetylated SOD2 protein expression and increased expression of SOD2 protein in THLE-2 and HEK293 cells suggest a protective mechanism against oxidative stress that was triggered 72 h post-treatment. Although BZD9L1 did not induce ROS production in HEK293 cells, other reactive oxygen and nitrogen species could elicit this response and thus warrant further investigation.

Apart from the three SIRTs (SIRT3–5) localised to mitochondria, suppression of SIRT1 by pan SIRT1 inhibitor has been associated with the induction of oxidative stress [57]. Reduction of cellular SIRT1 protein expression could lead to a reduction of antioxidant capacity and increased ROS and oxidative damage [58,59]. SIRT1 was shown to enhance the expression of MnSOD by deacetylating the p53 protein to increase cellular antioxidant capacity [58]. Nevertheless, acetylated p53 remained unaffected by BZD9L1 despite a reduction in SIRT1 protein expression. To our knowledge, it is unknown whether alteration of SIRT1 protein expression in the liver or kidney cells could lead to mitochondrial malfunction. In many cases, SIRT expressions are affected by the availability of substrates, which may also involve an intricate regulatory network and extensive feedback loop [60]. In this study, we showed that BZD9L1 modulates mitochondrial response toward oxidative stress in human-derived kidney cells by regulating SIRT1 and SIRT3 protein levels, which may then impact secondary mechanisms. However, a study focused on the molecular mechanism induced by BZD9L1 towards SIRT-intramitochondrial communication requires further delineation by exploring the (i) activity changes in each ETC complex, (ii) the impact on mitochondrial membrane potential, and (iii) the expression of mitochondrial stress markers. Although BZD9L1 had no impact on the spare respiratory capacity of the cells, further validation of mitochondrial function through assessment of other mitochondrial parameters is warranted to assess the safety of BZD9L1. This information may be crucial to explicate cellular behaviour changes in response to metabolic or oxidative stresses that may compromise mitochondrial function [61].

In a nutshell, the Seahorse XF Mito Stress Test offers a valuable advantage in the realm of drug discovery. Its real-time and highly sensitive assessment of mitochondrial function makes it an essential tool in the early stages of drug development. While the assay may not encompass the full range of metabolic processes or delve into the underlying mechanisms of action, it does provide initial insights into how a drug might influence mitochondrial function, guiding subsequent mechanistic investigations. It is worth noting that the assay relies on in vitro cell cultures, which may not fully replicate the intricate complexities of in vivo tissue microenvironment. To address this, integrated systems biology approaches that amalgamate the Seahorse XF Mito Stress Test findings with other -omics data (such as metabolomics, transcriptomics, and proteomics) can be instrumental in constructing comprehensive models of metabolic processes and their responses to BZD9L1. Moreover, the use of high-content imaging techniques is valuable for visualizing alterations in cellular and mitochondrial morphology induced by drugs. This approach sheds light on structural changes and their consequences for cellular function. More advanced technologies that utilise tissue chips or organ-on-a-chip techniques, such as microphysiological systems (MPS), may better emulate the human organ system in predicting outcomes in a more physiologically relevant manner [62]. Finally, evaluating BZD9L1 in vivo presents an opportunity for a more profound understanding of the compound’s impact on mitochondrial function and cellular metabolism within the context of drug discovery.

## 5. Conclusions

In this study, the in vitro model using normal human liver- and kidney-derived cell lines was employed to complement short-term toxicology evaluation in vivo. However, as in vitro cell culture lacks stromal cells and their interactions with the epithelial cells, further translation and interpretation from the former to the latter is therefore challenging. Nonetheless, measuring the OCR allows bioenergetic profiling to provide insights into how BZD9L1 may impact mitochondrial function. In short, BZD9L1 did not likely impair mitochondrial function in both cell lines as no changes were observed in the cellular spare respiratory capacity and ATP production despite an increase in proton leak and reduced coupling efficiency in THLE cells. As proton leak and coupling efficiency are not mutually exclusive, future assessment of membrane potential and the role of uncoupling protein (UCP) in regulating proton gradient may provide insights into the relationship between uncoupling efficiency and mitochondrial function upon BZD9L1 treatment. 

## Figures and Tables

**Table 1:** The IC_50_ of BZD9L1 in THLE-2 and HEK293 cells at 24, 48, and 72 h post-treatment.

Treatment Duration	Cell Viability (IC_50_), µM	
	THLE-2	HEK293
24 h	178.92 ± 0.049	77.72 ± 0.083
48 h	21.11 ± 0.052	31.00 ± 0.089
72 h	15.75 ± 0.051	23.74 ± 0.072

Abbreviations: h, hours; IC_50_, half-maximal inhibitory concentration.

## Data Availability

The authors confirm that the data supporting the findings of this study are available within its Supplementary Materials.

## Supplementary Materials

The following supporting information can be downloaded at https://www.mdpi.com/article/10.3390/biomedicines11113059/s1. Figure S1: Representative Western blots showing the protein levels of SIRT4 or SIRT5 in the THLE-2 and HEK293 cells upon BZD9L1 treatment at 24 h (lane 1–3) and 72 h (lane 4–6).

## References

[1] Mayr J.A. (2015). Lipid metabolism in mitochondrial membranes. J. Inherit. Metab. Dis..

[2] Piel R.B., Dailey H.A., Medlock A.E. (2019). The mitochondrial heme metabolon: Insights into the complex(ity) of heme synthesis and distribution. Mol. Genet. Metab..

[3] Roca-Agujetas V., de Dios C., Lestón L., Marí M., Morales A., Colell A. (2019). Recent Insights into the Mitochondrial Role in Autophagy and Its Regulation by Oxidative Stress. Oxid. Med. Cell. Longev..

[4] Will Y., Shields J.E., Wallace K.B. (2019). Drug-Induced Mitochondrial Toxicity in the Geriatric Population: Challenges and Future Directions. Biology.

[5] Palmeira C.M., Duarte F.V., Teodoro J.S., Varela A.T., Rolo A.P., Gupta R.C. (2014). Chapter 51*—*Biomarkers of mitochondrial dysfunction and toxicity. Biomarkers in Toxicology.

[6] Carafa V., Russo R., Della Torre L., Cuomo F., Dell’Aversana C., Sarno F., Sgueglia G., Di Donato M., Rotili D., Mai A. (2020). The Pan-Sirtuin Inhibitor MC2494 Regulates Mitochondrial Function in a Leukemia Cell Line. Front. Oncol..

[7] Lin Y.T., Lin K.H., Huang C.J., Wei A.C. (2021). MitoTox: A comprehensive mitochondrial toxicity database. BMC Bioinform..

[8] Dykens J.A., Will Y., Wexler P. (2014). Mitochondrial Toxicity. Encyclopedia of Toxicology.

[9] Wang R., Novick S.J., Mangum J.B., Queen K., Ferrick D.A., Rogers G.W., Stimmel J.B. (2015). The acute extracellular flux (XF) assay to assess compound effects on mitochondrial function. J. Biomol. Screen..

[10] Yépez V.A., Kremer L.S., Iuso A., Gusic M., Kopajtich R., Koňaříková E., Nadel A., Wachutka L., Prokisch H., Gagneur J. (2018). OCR-Stats: Robust estimation and statistical testing of mitochondrial respiration activities using Seahorse XF Analyzer. PLoS ONE.

[11] Brand M.D., Nicholls D.G. (2011). Assessing mitochondrial dysfunction in cells. Biochem. J..

[12] Jaber S.M., Yadava N., Polster B.M. (2020). Mapping mitochondrial respiratory chain deficiencies by respirometry: Beyond the Mito Stress Test. Exp. Neurol..

[13] Alhazzazi T.Y., Kamarajan P., Joo N., Huang J.-Y., Verdin E., D’Silva N.J., Kapila Y.L. (2011). Sirtuin-3 (SIRT3), a novel potential therapeutic target for oral cancer. Cancer.

[14] Lee Y.T., Tan Y.J., Mok P.Y., Subramaniam A.V., Oon C.E., Maiese K. (2021). Chapter 9*—*The bifunctional roles of sirtuins and their therapeutic potential in cancer. Sirtuin Biology in Cancer and Metabolic Disease.

[15] Huang J.Y., Hirschey M.D., Shimazu T., Ho L., Verdin E. (2010). Mitochondrial sirtuins. Biochim. Biophys. Acta.

[16] Tan Y.J., Lee Y.T., Yeong K.Y., Petersen S.H., Kono K., Tan S.C., Oon C.E. (2018). Anticancer activities of a benzimidazole compound through sirtuin inhibition in colorectal cancer. Future Med. Chem..

[17] Tan Y.J., Lee Y.T., Petersen S.H., Kaur G., Kono K., Tan S.C., Majid A.M.S.A., Oon C.E. (2019). BZD9L1 sirtuin inhibitor as a potential adjuvant for sensitization of colorectal cancer cells to 5-fluorouracil. Ther. Adv. Med. Oncol..

[18] Tan Y.J., Lee Y.T., Mancera R.L., Oon C.E. (2021). BZD9L1 sirtuin inhibitor: Identification of key molecular targets and their biological functions in HCT116 colorectal cancer cells. Life Sci..

[19] Lee Y.T., Tan Y.J., Mok P.Y., Kaur G., Sreenivasan S., Falasca M., Oon C.E. (2022). Sex-divergent expression of cytochrome P450 and SIRTUIN 1-7 proteins in toxicity evaluation of a benzimidazole-derived epigenetic modulator in mice. Toxicol. Appl. Pharmacol..

[20] Zhong L., Mostoslavsky R. (2011). Fine tuning our cellular factories: Sirtuins in mitochondrial biology. Cell Metab..

[21] Fanibunda S.E., Deb S., Maniyadath B., Tiwari P., Ghai U., Gupta S., Figueiredo D., Weisstaub N., Gingrich J.A., Vaidya A.D.B. (2019). Serotonin regulates mitochondrial biogenesis and function in rodent cortical neurons via the 5-HT_2A_ receptor and SIRT1—PGC-1α axis. Proc. Natl. Acad. Sci. USA.

[22] Kwon S.J., Lee D.W., Shah D.A., Ku B., Jeon S.Y., Solanki K., Ryan J.D., Clark D.S., Dordick J.S., Lee M.-Y. (2014). High-throughput and combinatorial gene expression on a chip for metabolism-induced toxicology screening. Nat. Commun..

[23] Capes-Davis A., Bairoch A., Barrett T., Burnett E.C., Dirks W.G., Hall E.M., Healy L., Kniss D.A., Korch C., Liu Y. (2019). Cell Lines as Biological Models: Practical Steps for More Reliable Research. Chem. Res. Toxicol..

[24] Guo L., Dial S., Shi L., Branham W., Liu J., Fang J.L., Green B., Deng H., Kaput J., Ning B. (2011). Similarities and differences in the expression of drug-metabolizing enzymes between human hepatic cell lines and primary human hepatocytes. Drug Metab. Dispos..

[25] Little A.C., Kovalenko I., Goo L.E., Hong H.S., Kerk S.A., Yates J.A., Purohit V., Lombard D.B., Merajver S.D., Lyssiotis C.A. (2020). High-content fluorescence imaging with the metabolic flux assay reveals insights into mitochondrial properties and functions. Commun. Biol..

[26] Lee J.T., Gu W. (2013). SIRT1: Regulator of p53 Deacetylation. Genes Cancer.

[27] Eakins J., Bauch C., Woodhouse H., Park B., Bevan S., Dilworth C., Walker P. (2016). A combined in vitro approach to improve the prediction of mitochondrial toxicants. Toxicol. Vitr. Int. J. Publ. Assoc. BIBRA.

[28] Rana P., Aleo M.D., Gosink M., Will Y. (2019). Evaluation of in Vitro Mitochondrial Toxicity Assays and Physicochemical Properties for Prediction of Organ Toxicity Using 228 Pharmaceutical Drugs. Chem. Res. Toxicol..

[29] Cortassa S., O’Rourke B., Aon M.A. (2014). Redox-Optimized ROS Balance and the relationship between mitochondrial respiration and ROS. Biochim. Et Biophys. Acta (BBA)—Bioenerg..

[30] Nanayakkara G.K., Wang H., Yang X. (2019). Proton leak regulates mitochondrial reactive oxygen species generation in endothelial cell activation and inflammation*—*A novel concept. Arch. Biochem. Biophys..

[31] Dranka B.P., Benavides G.A., Diers A.R., Giordano S., Zelickson B.R., Reily C., Zou L., Chatham J.C., Hill B.G., Zhang J. (2011). Assessing bioenergetic function in response to oxidative stress by metabolic profiling. Free. Radic. Biol. Med..

[32] Gnaiger E., Boushel R., Søndergaard H., Munch-Andersen T., Damsgaard R., Hagen C., Díez-Sánchez C., Ara I., Wright-Paradis C., Schrauwen P. (2015). Mitochondrial coupling and capacity of oxidative phosphorylation in skeletal muscle of Inuit and Caucasians in the arctic winter. Scand. J. Med. Sci. Sports.

[33] Cheng J., Nanayakkara G., Shao Y., Cueto R., Wang L., Yang W.Y., Tian Y., Wang H., Yang X. (2017). Mitochondrial Proton Leak Plays a Critical Role in Pathogenesis of Cardiovascular Diseases. Adv. Exp. Med. Biol..

[34] Demine S., Renard P., Arnould T. (2019). Mitochondrial Uncoupling: A Key Controller of Biological Processes in Physiology and Diseases. Cells.

[35] Hill B.G., Benavides G.A., Lancaster J.R., Ballinger S., Dell’Italia L., Jianhua Z., Darley-Usmar V.M. (2012). Integration of cellular bioenergetics with mitochondrial quality control and autophagy. Biol. Chem..

[36] Jastroch M., Divakaruni A.S., Mookerjee S., Treberg J.R., Brand M.D. (2010). Mitochondrial proton and electron leaks. Essays Biochem..

[37] Marchetti D., Reiland J., Erwin B., Roy M. (2003). Inhibition of heparanase activity and heparanase-induced angiogenesis by suramin analogues. Int. J. Cancer.

[38] Marchetti P., Fovez Q., Germain N., Khamari R., Kluza J. (2020). Mitochondrial spare respiratory capacity: Mechanisms, regulation, and significance in non-transformed and cancer cells. FASEB J..

[39] Pour P.A., Kenney M.C., Kheradvar A. (2020). Bioenergetics Consequences of Mitochondrial Transplantation in Cardiomyocytes. J. Am. Heart Assoc..

[40] Will Y., Dykens J. (2014). Mitochondrial toxicity assessment in industry--a decade of technology development and insight. Expert Opin Drug Metab. Toxicol..

[41] van der Stel W., Carta G., Eakins J., Darici S., Delp J., Forsby A., Bennekou S.H., Gardner I., Leist M., Danen E.H.J. (2020). Multiparametric assessment of mitochondrial respiratory inhibition in HepG2 and RPTEC/TERT1 cells using a panel of mitochondrial targeting agrochemicals. Arch. Toxicol..

[42] Divakaruni A.S., Paradyse A., Ferrick D.A., Murphy A.N., Jastroch M., Murphy A.N., Chan D.C. (2014). Chapter Sixteen*—*Analysis and Interpretation of Microplate-Based Oxygen Consumption and pH Data. Methods in Enzymology.

[43] Chan G.K., Kleinheinz T.L., Peterson D., Moffat J.G. (2013). A simple high-content cell cycle assay reveals frequent discrepancies between cell number and ATP and MTS proliferation assays. PLoS ONE.

[44] Kam Y., Rogers G.W., Jastromb N., Dranka B.P. Methods and Strategies for Normalizing XF Metabolic Data to Cellular Parameters. https://www.agilent.com/cs/library/technicaloverviews/public/Methods_and_Strategies_for_Normalizing_Tech_Overview_022118.pdf.

[45] de Moura M.B., Uppala R., Zhang Y., Van Houten B., Goetzman E.S. (2014). Overexpression of mitochondrial sirtuins alters glycolysis and mitochondrial function in HEK293 cells. PLoS ONE.

[46] Lombard D.B., Alt F.W., Cheng H.-L., Bunkenborg J., Streeper R.S., Mostoslavsky R., Kim J., Yancopoulos G., Valenzuela D., Murphy A. (2007). Mammalian Sir2 homolog SIRT3 regulates global mitochondrial lysine acetylation. Mol. Cell. Biol..

[47] Pfeifer A.M., Cole K.E., Smoot D.T., Weston A., Groopman J.D., Shields P.G., Vignaud J.M., Juillerat M., Lipsky M.M., Trump B.F. (1993). Simian virus 40 large tumor antigen-immortalized normal human liver epithelial cells express hepatocyte characteristics and metabolize chemical carcinogens. Proc. Natl. Acad. Sci. USA.

[48] Papa L., Germain D. (2014). SirT3 regulates the mitochondrial unfolded protein response. Mol. Cell Biol..

[49] Li H., Cai Z. (2022). SIRT3 regulates mitochondrial biogenesis in aging-related diseases. J. Biomed. Res..

[50] Buler M., Aatsinki S.-M., Izzi V., Hakkola J. (2012). Metformin Reduces Hepatic Expression of SIRT3, the Mitochondrial Deacetylase Controlling Energy Metabolism. PLoS ONE.

[51] Kokoszka J.E., Coskun P., Esposito L.A., Wallace D.C. (2001). Increased mitochondrial oxidative stress in the Sod2 (+/−) mouse results in the age-related decline of mitochondrial function culminating in increased apoptosis. Proc. Natl. Acad. Sci. USA.

[52] Chen Y., Zhang J., Lin Y., Lei Q., Guan K.L., Zhao S., Xiong Y. (2011). Tumour suppressor SIRT3 deacetylates and activates manganese superoxide dismutase to scavenge ROS. EMBO Rep..

[53] Candas D., Li J.J. (2014). MnSOD in oxidative stress response-potential regulation via mitochondrial protein influx. Antioxid. Redox Signal..

[54] Wang Y., Branicky R., Noë A., Hekimi S. (2018). Superoxide dismutases: Dual roles in controlling ROS damage and regulating ROS signaling. J. Cell Biol..

[55] Shen Y., Wu Q., Shi J., Zhou S. (2020). Regulation of SIRT3 on mitochondrial functions and oxidative stress in Parkinson’s disease. Biomed. Pharmacother. Biomed. Pharmacother..

[56] Hjelmeland A.B., Patel R.P. (2019). SOD2 acetylation and deacetylation: Another tale of Jekyll and Hyde in cancer. Proc. Natl. Acad. Sci. USA.

[57] Wan X., Garg N.J. (2021). Sirtuin Control of Mitochondrial Dysfunction, Oxidative Stress, and Inflammation in Chagas Disease Models. Front. Cell. Infect. Microbiol..

[58] Ren Z., He H., Zuo Z., Xu Z., Wei Z., Deng J. (2019). The role of different SIRT1-mediated signaling pathways in toxic injury. Cell. Mol. Biol. Lett..

[59] Alam F., Syed H., Amjad S., Baig M., Khan T.A., Rehman R. (2021). Interplay between oxidative stress, SIRT1, reproductive and metabolic functions. Curr. Res. Physiol..

[60] Buler M., Andersson U., Hakkola J. (2016). Who watches the watchmen? Regulation of the expression and activity of sirtuins. FASEB J..

[61] Takahashi E., Yamaoka Y. (2017). Simple and inexpensive technique for measuring oxygen consumption rate in adherent cultured cells. J. Physiol. Sci..

[62] Cirit M., Stokes C.L. (2018). Maximizing the impact of microphysiological systems with in vitro-in vivo translation. Lab Chip.

