# Supplementary material for: BZD9L1 Differentially Regulates Sirtuins in Liver-Derived Cells by Inducing Reactive Oxygen Species
Source: Biomedicines. 2023 Nov 15;11(11):3059. doi: 10.3390/biomedicines11113059 (PMC10669747; doi:10.3390/biomedicines11113059)
Supplement: Supplementary file 1 [file biomedicines-11-03059-s001.zip › biomedicines-2413191-supplementary.pdf]

Supplementary Figure

a) THLE-2

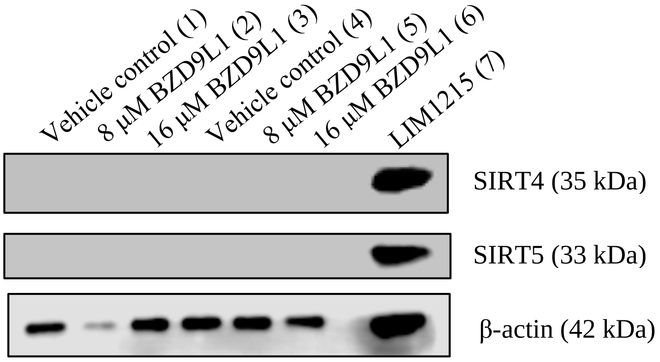

b) HEK293

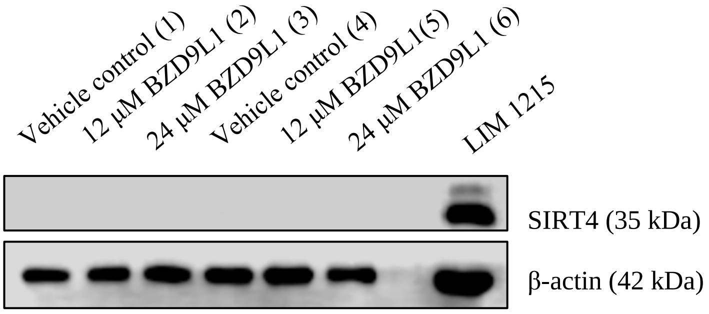

Supplementary Figure S1. Representative western blots showing proteins levels of SIRT4 and SIRT5 in THLE-2 and HEK293 cells upon BZD9L1 treatment for 24 h (lane 1–3) and 72 h (lane 4–6), n = 3 independent experiments. β-actin was used as a loading control. LIM1215 colorectal cancer cells were used as a positive control to confirm the validity of the SIRT4 and SIRT5 antibodies.
